# Evolution of atomic structure during nanoparticle formation

**DOI:** 10.1107/S2052252514006538

**Published:** 2014-04-14

**Authors:** Christoffer Tyrsted, Nina Lock, Kirsten M. Ø. Jensen, Mogens Christensen, Espen D. Bøjesen, Hermann Emerich, Gavin Vaughan, Simon J. L. Billinge, Bo B. Iversen

**Affiliations:** a Center for Materials Crystallography, Department of Chemistry, and iNANO, Aarhus University, Langelandsgade 140, Aarhus, DK-8000, Denmark; b Faculty of Chemistry, Georg-August-Universitat Gottingen, Tammannstrasse 4, D-37077 Gottingen, Germany; c Department of Applied Physics and Applied Mathematics, Columbia University, New York, NY 10027, USA; d SNBL, European Synchrotron Radiation Facility, 6 rue Horowitz, F-38043 Grenoble, France; e ID11, European Synchrotron Radiation Facility, 6 rue Horowitz, F-38043 Grenoble, France; f Condensed Matter Physics and Materials Science Department, Brookhaven National Laboratory, Upton, New York, NY 11973, USA

**Keywords:** total scattering, EXAFS, PDF, *in situ*, nanoparticle

## Abstract

Understanding the mechanism of nanoparticle formation during synthesis is a key prerequisite for the rational design and engineering of desirable materials properties, yet remains elusive due to the difficulty of studying structures at the nanoscale under real conditions. Here, the first comprehensive structural description of the formation of a nanoparticle, yttria-stabilized zirconia (YSZ), all the way from its ionic constituents in solution to the final crystal, is presented. The transformation is a complicated multi-step sequence of atomic reorganizations as the material follows the reaction pathway towards the equilibrium product. Prior to nanoparticle nucleation, reagents reorganize into polymeric species whose structure is incompatible with the final product. Instead of direct nucleation of clusters into the final product lattice, a highly disordered intermediate precipitate forms with a local bonding environment similar to the product yet lacking the correct topology. During maturation, bond reforming occurs by nucleation and growth of distinct domains within the amorphous intermediary. The present study moves beyond kinetic modeling by providing detailed real-time structural insight, and it is demonstrated that YSZ nanoparticle formation and growth is a more complex chemical process than accounted for in conventional models. This level of mechanistic understanding of the nanoparticle formation is the first step towards more rational control over nanoparticle synthesis through control of both solution precursors and reaction intermediaries.

## Abstract 2

The complete structural transformation of ionic species in the precursor solution, over an amorphous solid and finally into crystalline nanoparticles, is elucidated by *in situ* investigations under supercritical solvothermal conditions.

## 1. Introduction   

Nanoparticles have unique properties different from bulk crystals, forming the core of numerous modern technologies (Aricò *et al.*, 2005 ▶). In this context, solvothermal synthesis has emerged as one of the preferred approaches for controlled preparation of technologically important inorganic nano­materials on both laboratory and industrial scale (Adschiri *et al.*, 1992 ▶; Savage *et al.*, 1995 ▶; Walton, 2002 ▶; Aymonier *et al.*, 2006 ▶). Among the important modern-day materials preferably produced through solvothermal processes is yttria-stabilized zirconia (YSZ), investigated here. The material has attracted immense interest in both academia and industry, exhibiting one of the highest known oxide ion conductivities, making it the material of choice for commercial solid oxide fuel cell membranes (Goodenough, 2003 ▶; Fergus, 2006 ▶; Hua *et al.*, 2006 ▶; Tyrsted *et al.*, 2012*a* ▶). Here, as for all other materials, the insight into formation is of utmost importance for controlled material design. Understanding material formation from solution therefore remains as one of the key challenges in material science. In general, the solvothermal formation of materials may be divided into two distinct stages, pre-nucleation and post-nucleation. The post-nucleation period of nanoparticle formation is more easily accessible due to the presence of well defined and stable structural units (Pienack & Bensch, 2011 ▶; Walton & Hare, 2000 ▶; Millange *et al.*, 2010 ▶; Lock *et al.*, 2009 ▶; Cheong *et al.*, 2009 ▶; Zhang *et al.*, 2007 ▶). Nonetheless, the more elusive pre-nucleation stages have attracted increased focus owing to newly developed experimental methods (Jensen *et al.*, 2012 ▶; Tyrsted *et al.*, 2012*b* ▶; Ok *et al.*, 2012 ▶; Chupas *et al.*, 2007 ▶). Still, the structural understanding of formation processes has been restricted to investigating isolated structural stages rather than describing the apparently chaotic transformation steps. Improving this understanding, we present the first complete atomic-level description of the formation of a nanoparticle (YSZ) from its molecular precursors, spanning both pre- and post-nucleation periods, revealing a complex multi-step formation pathway. We show that the processes involved in the solvothermal synthesis are evidently more complex than the simple crystallization mechanisms often assumed in classical kinetics modeling, demonstrating the importance of structural understanding. This comprehensive description is made possible by combining *in situ* total scattering (TS) and pair distribution function (PDF) analysis with *in situ* X-ray absorption spectroscopy (XAS) and extended X-ray absorption fine-structure (EXAFS) analysis.

## 2. Experimental   

### 2.1. Total X-ray scattering   

#### 2.1.1. Total scattering measurements   

*In situ* total scattering experiments were performed at beamline ID11 at the European Synchrotron Radiation Facility, Grenoble, France. The synthesis precursor was prepared by dissolving the appropriate amount of ZrO(NO_3_)_2_·6H_2_O (Sigma Aldrich, >98%) and Y(NO_3_)_3_·4H_2_O (Sigma Aldrich, >98%) in methanol (99%) for an intended molar substitution of 8% Y_2_O_3_ into the ZrO_2_ lattice. The [Zr^4+^] + [Y^3+^] molar concentration of 1 *M* yielded a colorless translucent solution. The syntheses were performed by loading the precursor into a 0.6/0.7 mm-diameter (inner/outer) fused silica capillary. The capillary was pressurized to 230 bar by a LabAlliance HPLC pump using methanol (99%) as the pressurizing medium. Afterwards, the capillary was heated to 548 K by a hot air jet with temperature measured by a K-type thermocouple. The supercritical point of pure methanol is *T*_crit_ = 512 K and *P*_crit_ = 80 bar. Owing to the small volume of the capillary and the efficiency of the heater, 90% of the set-point temperature was reached within the first 10 s of heating (Becker *et al.*, 2010 ▶). Concurrent with initiation of the heating, scattering from a monochromatic X-ray beam (λ = 0.1897 Å) was recorded by a Perkin Elmer a-Si flat-panel detector. The sample-to-detector distance was 227 mm, making it possible to reach a *Q*_max_ of around 21 Å^−1^, where *Q* is the magnitude of the scattering vector, *Q* = 4πsinθ/λ. Detector frames were read out with a time resolution of 2.5 s. This was chosen as the best compromise between data quality and time resolution. Representative detector images may be seen in Fig. S1 of the supporting information, clearly illustrating the difference in total scattering observed during different stages of the synthesis.

#### 2.1.2. PDF analysis   

The two-dimensional detector frames obtained from the total scattering experiments were integrated using the *FIT2D* software (Hammersley *et al.*, 1996 ▶). Prior to integration, the X-ray wavelength was calibrated, as was the detector geometry such as distance from the sample and obliquity, using a LaB_6_ (NIST) sample.

The integrated total scattering data were analyzed using the PDF method (Egami & Billinge, 2012 ▶). The reduced pair distribution functions, *G*(*r*), were obtained from the integrated data using the *PDFgetX3* program (Juhás *et al.*, 2013 ▶). Prior to the Fourier transform, integrated data were corrected for background scattering using measurements on methanol in the same capillary at comparable temperatures. Structure functions were used up to a *Q*_max_ of 17 Å^−1^, where the *Q*-range was limited from the theoretical maximum in this case by signal-to-noise issues after subtracting the background of pure methanol. This limits the PDF resolution to around 0.2 Å, making it difficult to distinguish overlapping correlation peaks within this distance (Proffen, 2012 ▶). The resulting PDFs were refined sequentially in *PDFfit2* using *PDFgui* (Farrow *et al.*, 2007 ▶). The structural refinement of crystalline Y_0.16_Zr_0.84_O_2-d_ (Table S1) is based on crystallographic data of the cubic lattice structure of Y_0.2_Zr_0.8_O_1.9_ from ICSD-75316 (Yashima *et al.*, 1994 ▶). The structural refinement of the amorphous matrix (Table S2) is based on crystallographic data of monoclinic ZrO_2_ from ICSD-18190 (Smith & Newkirk, 1965 ▶). The structural refinement of the aqueous precursor solution (Table S3 and Fig. S3) is based on a modified structural model obtained from the crystallographic data of Zr(OH)_2_(NO_3_)_2_(H_2_O)_4.71_ (ICSD-80603) (Bénard *et al.*, 1991 ▶). The instrumental resolution was determined from a NIST LaB_6_ standard yielding a *Q*_damp_ of 0.028945 Å^−1^ (Farrow *et al.*, 2007 ▶). The change in the shortest metal–oxygen and metal–metal distances (Fig. 4) during transformation was followed using single peak fits to the respective PDF correlation peaks. Significant noise contributions to the *r* < 1.5 Å region of the PDFs are caused by inadequacies in the data corrections and truncation errors. This region is, however, not structurally relevant as the shortest metal–oxygen distances start above 2.0 Å. A visual comparison of the structural refinement of all three stages may be observed in Fig. S3. The parameter uncertainties were obtained through a refinement carried out on a Nyqvist–Shannon sampling grid (Farrow *et al.*, 2011 ▶).

### 2.2. X-ray absorption spectroscopy   

#### 2.2.1. X-ray absorption spectroscopy measurements   

*In situ* X-ray absorption experiments were performed at beamline BM01B (ESRF, Grenoble, France) in transmission mode using a set-up consisting of three ionization chambers. Precursor preparation and synthesis conditions equal that for the total scattering experiments.

Measurements on the local environment of Zr and Y were performed as two identical synthesis experiments probing the Zr *K*-edge and Y *K*-edge, respectively. The absorption spectroscopy of the Zr *K*-edge was performed as a continuous scan in the range 17.85–18.90 keV while the Y *K*-edge was measured in the energy range 16.95–17.75 keV. Powders of ZrO(NO_3_)_2_·6H_2_O and Y(NO_3_)_3_·4H_2_O were used as references for measurements at the corresponding absorption edges. The time-resolution of each energy scan was 20 s.

#### 2.2.2. EXAFS analysis   

Data processing was performed using the software program *WinXAS* (Ressler, 1998 ▶). The background was subtracted using a first-order polynomial fit to the pre-edge region, and data were normalized to eliminate the effect of sample thickness. For calibration, the edge energy was chosen as the zero-crossing of the second-derivate. The EXAFS signal was isolated from the normalized spectrum by subtracting the absorption from an isolated absorber atom determined by fitting a smooth background to the measured spectrum using a cubic splines function with ten splines in the range ∼2.0–10.0 Å^−1^. The range of the isolated EXAFS signal was limited with a Bessel window in the range ∼2.5–9.0 Å^−1^ and Fourier transformed to obtain a phase-shifted radial distribution function in real space.

Atomic scattering paths were generated by the *Atoms* (Ravel, 2001 ▶) and *Feff* (Rehr *et al.*, 2010 ▶) codes based on the crystallographic data from ICSD-75316 (Yashima *et al.*, 1994 ▶). Data fitting (in *R*-space) of the two nearest coordination shells in the range 1–4 Å was performed in *WinXAS*, allowing for a Nyquist number of independent parameters of ∼14 (Stern, 1993 ▶). Normalized absorption edges, *k*-space data and data analysis examples may be seen in Figs. S4, S5 and S6.

## 3. Results and discussion   

**Figure 1:**
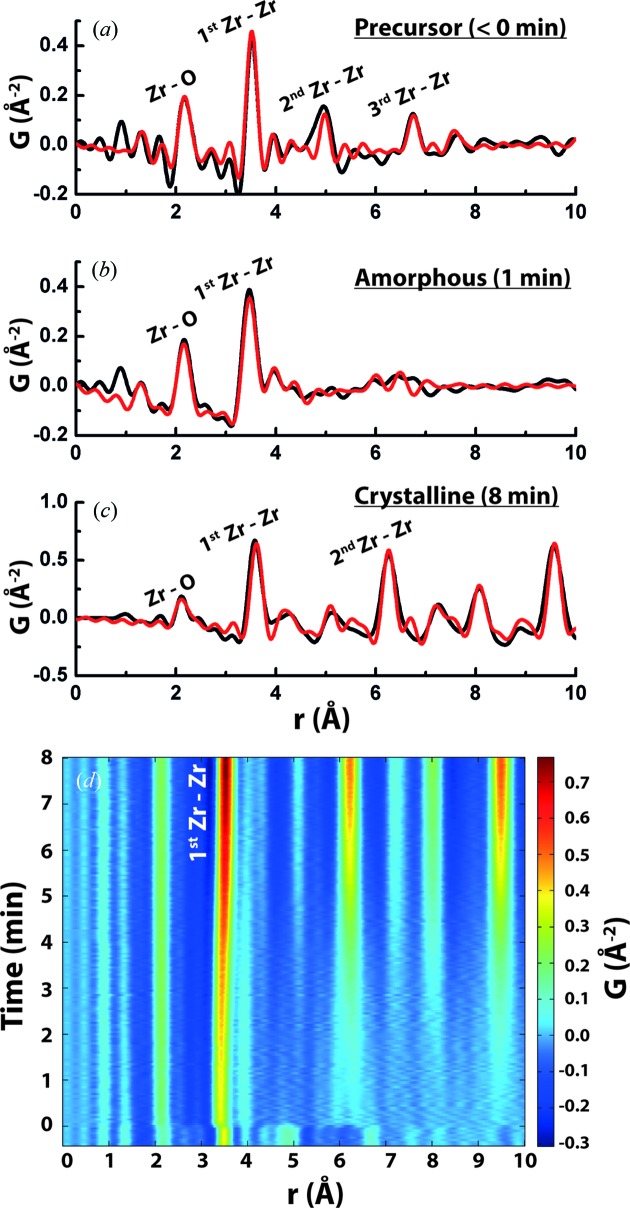
Local atomic ordering as revealed by total scattering. PDF (black line) and structural modeling (red line) for (*a*) the precursor solution prior to nucleation, (*b*) the amorphous precipitates formed after nucleation and (*c*) nanocrystalline domains present after prolonged reaction. (*d*) Time-resolved view of the local structural region (0–10 Å) of the PDF.

The solvothermal formation of YSZ (8 mol% Y_2_O_3_) nanoparticles may be described as the transformation of an ionic solution of Zr^4+^ and Y^3+^ species into pristine crystalline nanoparticles within a few minutes at moderate temperatures (548 K). The detailed transformation mechanism is revealed in our measurements of the local atomic structure as the material evolves through the process from pre-nucleation to post-nucleation. Characteristic atomic PDFs (Egami & Billinge, 2012 ▶) from X-ray total scattering measurements corresponding to various stages of the synthesis are shown in Fig. 1 ▶. They are strikingly different, implying distinct structural stages during synthesis. Considering the pre-nucleation period (time *t* < 0 min), the PDF exhibits four clearly resolved peak features at 2.3 Å, 3.6 Å, 4.9 Å and 6.7 Å. The first peak can be assigned to the chemical bonding of Zr—O, while the remainder may be assigned to metal–metal distances based on their strong intensity. Here, the observation of the pronounced peak at 6.7 Å unambiguously indicates the presence of reasonably well ordered precursor clusters in solution, in contrast to ligated species of single metal ions.

Upon initiation of synthesis, through applied heating (548 K at *t* = 0), there is a rapid change in the observed PDF (Fig. 1*b* ▶). The most striking aspect of this PDF is the loss of structural order beyond the immediate nearest (1st) neighbor region. This could be caused by a breakdown of polymeric precursors into smaller clusters. However, as known from small-angle scattering, large clusters are precipitated during this stage of the synthesis (Tyrsted *et al.*, 2012*a* ▶). Therefore, the PDF signal is originating from relatively large precipitated clusters which are highly disordered and amorphous in nature. The two surviving interatomic distances at around 2.2 Å and 3.5 Å (Fig. 1 ▶ *b*) are significantly shorter than those of the precursor, yet resemble the nearest (1st) neighbor and second-nearest (2nd) neighbor distances in the mature YSZ product emerging after 8 min of reaction (Fig. 1*c* ▶). As a combined process (Fig. 1*d*), the rapid bond-shortening, loss of structural order and gradual reappearance of PDF peaks in the intermediate distance range are very apparent as the reaction evolves. The formation of YSZ nanoparticles during solvothermal synthesis therefore appears to exhibit three distinct structural stages: precursor species, amorphous solid and ordered nanocrystalline solid.

**Figure 2:**
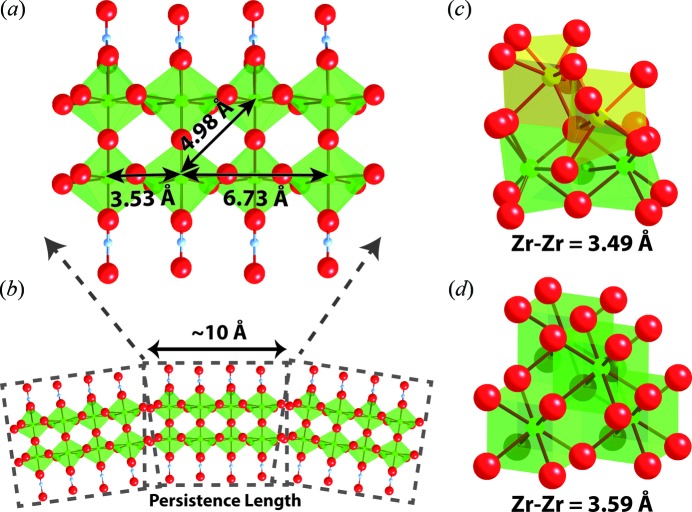
Structural stages observed during yttria-stabilized zirconia formation. (*a*) Structural model of the zirconia double-chain existing in the precursor solution part of a (*b*) polymeric chain. (*c*) Amorphous structure formed after precipitation containing distinct rigid units (yellow). (*d*) Mature cubic crystalline structure of YSZ. Oxygen: red; zirconium: green and yellow; nitrogen: blue. Yttrium is here structurally equivalent to zirconium.

The structure of the different material stages was further investigated through detailed modeling of the experimental PDFs. The local order observed in the pre-nucleation stage corresponds to the existence of previously unknown double-chained zirconia polymers (Fig. 2*a* ▶). Each individual chain is twisted along its length with a Zr—Zr—Zr angle of 145° giving rise to a nearest Zr—Zr distance of 3.53 Å and a third-nearest Zr—Zr distance of 6.73 Å. The combination of two single chains explains the existence of a second-nearest (2nd) neighbor Zr—Zr distance of 4.98 Å. The reduction of the PDF peak intensities with increasing *r* corresponds to an intermediate range order along the length of the chain modeled to be around 10 Å. This length may be described as an average persistence length over which the double-chain polymer appears rigid. The polymer structure is believed to survive beyond this persistence length (Bremholm *et al.*, 2014 ▶), but its flexibility results in a loss of well defined structural correlations due to large amplitude librational motions (Fig. 2*b* ▶).

The intermediate range order is lost upon transformation of the polymeric chains into the amorphous phase during precipitation. In the amorphous phase, our modeling is consistent with the presence of well defined [(OH)_*x*_(O)_4–*x*_Zr—O_2_—Zr(O)_4–*x*_(OH)_*x*_] structural units (yellow polyhedra, Fig. 2*c* ▶) having nearest (1st) neighbor Zr—Zr distances of 3.49 Å, yet with a complete loss of order over longer length scales. The amorphous structure could be described through the local structure of monoclinic (*P*2_1_/*c*) zirconia with a maximum correlation distance of around 8 Å corresponding to approximately one to two monoclinic unit cells. The refined oxygen occupancy for the structure is around ∼86% of that expected for sevenfold coordination, suggesting that the actual oxygen coordination of zirconium is closer to six. Furthermore, the nearest (1st) neighbor Zr—Zr peak is well defined and close in position to the crystalline YSZ case, though the relative integrated intensity is strongly suppressed with respect to the Zr—O correlation peak suggesting increased disorder compared with the crystalline structure. Nonetheless, the local environment of the rigid polyhedral units resembles that of the final product with connections between different polyhedral units being ill-defined, hindering the structure in propagating throughout the precipitate. We argue that the resultant need for reordering bonds between polyhedral units explains the slow reorganization of the amorphous structure, given the energy barrier associated with breaking and reforming bonds. The expected cubic (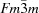) fluorite structure (Fig. 2*d*) yields a good structural description of the final observed PDF (Fig. 1*c* ▶), with a final nearest (1st) neighbor Zr—Zr distance of around 3.59 Å, corresponding to an average expansion of around 10 pm going from amorphous to crystalline material.

**Figure 3:**
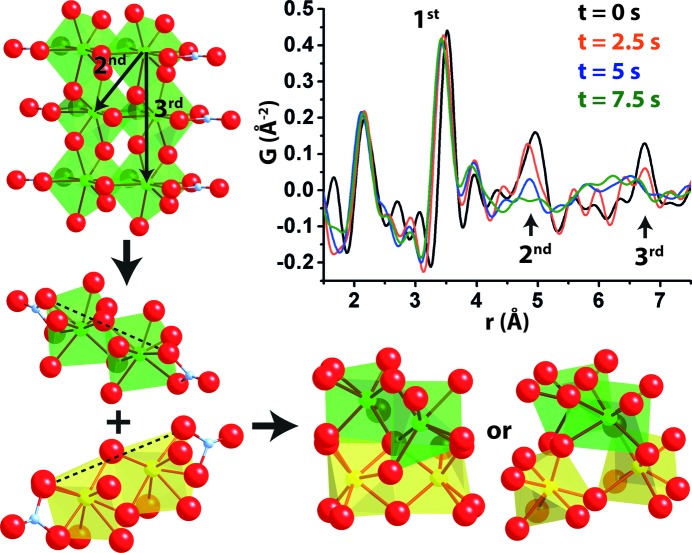
Initial nucleation mechanism. Total scattering local environment PDF and visualized transformation route of polymeric precursor species into amorphous matrix. Zirconium polyhedra are colored green and yellow with dotted lines indicating cluster orientation.

During the transformation from polymeric chains to amorphous precipitates, a rapid loss of the third-nearest (3rd) neighbor peak is followed by a disappearance of the second-nearest (2nd) Zr—Zr correlation as observed in Fig. 3 ▶. The polymers may therefore be understood as fragmenting along their length, before forming an amorphous precipitate. Following fragmentation, the low solubility of the solvothermal fluid forces a clustering of the fragmented double polyhedra into amorphous precipitates. The polyhedra may combine in two possible local morphologies found from the amorphous structure (Fig. 3 ▶) and it is evident that the local amorphous arrangement closely resembles that of the fragmented clusters. There is therefore little structural hindrance in forming the amorphous phase compared with the direct assembly of the cubic (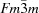) lattice.

**Figure 4:**
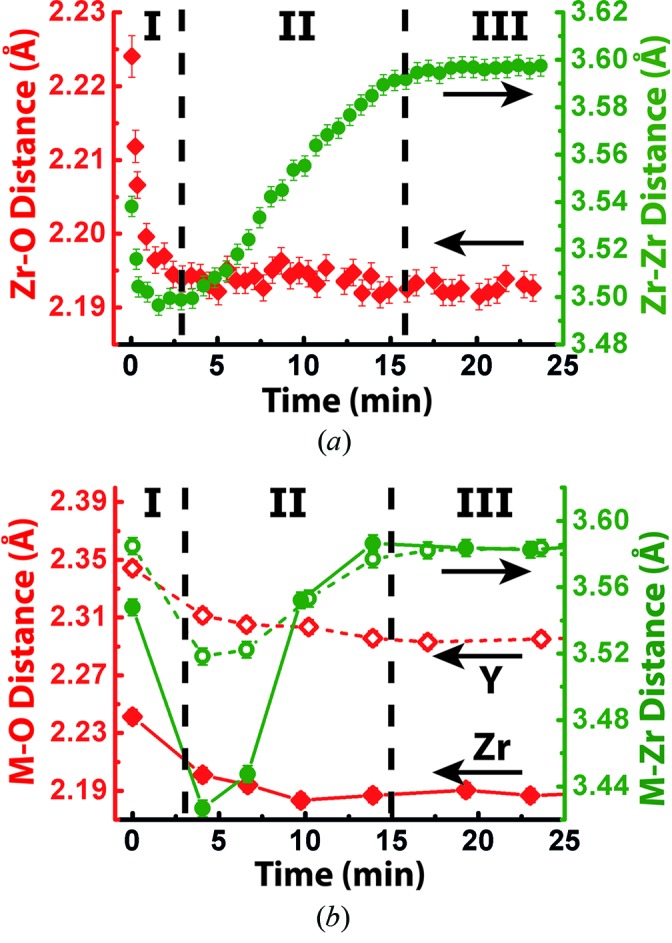
Changing local environment of zirconium and yttrium as observed by total scattering PDF and EXAFS. (*a*) Change in the two shortest interatomic distances as obtained through total scattering PDF analysis. For clarity, only every 15th datapoint is shown. (*b*) Change in the two shortest interatomic distances as obtained through EXAFS analysis. Dotted lines correspond to the change in the local structure surrounding yttrium, while solid lines correspond to the local structure of zirconium. Explanations of the stages I, II and III may be found in the text.

Following fragmentation, the local bond lengths rapidly contract from 2.23 Å to 2.20 Å for the Zr—O bond and 3.53 Å to 3.49 Å for the nearest (1st) neighbor Zr—Zr distance (Stage I, Fig. 4*a* ▶). This contraction likely originates from a positive pressure exerted on the isolated double-polyhedra by the low-solubility solvothermal fluid. As the amorphous matrix matures, the Zr—O bond distance remains close to constant whereas there is a significant expansion of the Zr—Zr distance from 3.49 Å to 3.59 Å (Stage II, Fig. 4*a* ▶). After the initial maturation, a stable stage is reached with little local structural change as bond reforming has been completed between all double polyhedra (Stage III, Fig. 4*a* ▶). The structural path of Zr^4+^ and Y^3+^ may be understood individually by probing the different atomic absorption edges (Fig. 4*b*). The change in interatomic distances for Zr—O and nearest (1st) neighbor Zr—Zr as observed by EXAFS analysis is close to that obtained through total scattering PDF analysis. Moreover, the change in the local environment of Y is similar to that observed for Zr. The initial nearest (1st) neighbor Y—*M* (*M* = Y or Zr) distance for the pre-nucleation stage is close to equal to the initial Zr—*M* distance indicating that Y^3+^ is part of the polymeric Zr^4+^ structure prior to nucleation. Upon nucleation, the Y—Zr distance contracts slightly less than the Zr—Zr distance to around 3.52 Å, as it is restricted by the larger ionic radius of Y^3+^ (0.90 Å, octahedral coordination) compared with Zr^4+^ (0.72 Å, octahedral coordination) (Shannon, 1976 ▶). As the amorphous matrix crystallizes, the nearest (1st) neighbor Y—Zr distance expands to 3.59 Å, equal to the distance for the nearest (1st) neighbor Zr—Zr showing that yttrium is ordering together with zirconium into the cubic lattice. The difference between the final Y—O and Zr—O distances is in agreement with *ex situ* EXAFS measurements by Rush *et al.* (2000 ▶). This indicates that yttrium is an inherent part of all structural stages and not inserted into the crystalline zirconia lattice at a later stage through a substitution process.

**Figure 5:**
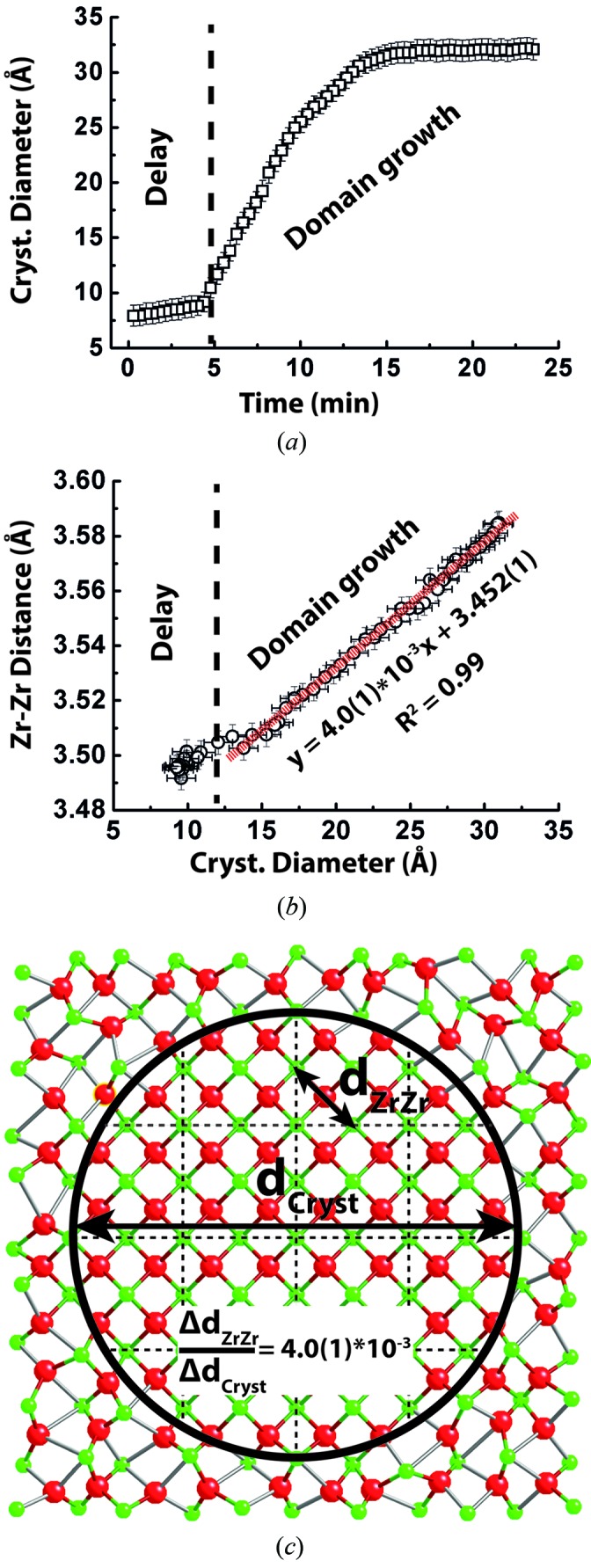
Post-nucleation structural transformation of amorphous precipitates. (*a*) Crystallite diameter growth curve obtained from total scattering. (*b*) Expansion of the nearest Zr—Zr distance as a function of growth in the coherent domain diameter. (*c*) Depiction of the size regime of individual crystalline domains.

The growth of individual crystalline domains from within the amorphous precipitate (Fig. 5*a* ▶) can be observed directly from the PDF by modeling the range of *r* over which correlations are seen in the final product (Egami & Billinge, 2012 ▶). After a few minutes latency, the range of structural coherence gradually increases, saturating at around 3.5 nm after 15 min thereby indicating complete ordering of the amorphous precipitate into crystalline nanodomains. During this process, the evolution of the nearest (1st) neighbor Zr—Zr distance remains a single PDF correlation peak showing that the local ordering of polyhedra is unchanged throughout the material. However, the expansion of the nearest (1st) neighbor Zr—Zr distance is closely related to the growth of the crystalline domains (Fig. 5*b* ▶) as evident by a linear correlation between crystal diameter and Zr—Zr distance (Fig. 5*c* ▶). The increase corresponds to a nearest (1st) neighbor Zr—Zr expansion of around 4 pm for each nanometer of growth in coherent domain diameter (Fig. 5*c* ▶). Again, this expansion may be related to the ordering of the oxygen sublattice during bond reforming having larger spatial requirements in the eightfold coordinated cubic lattice compared with the lower coordination observed in the amorphous matrix.

Particle nucleation and crystallization in solvothermal synthesis are often investigated through kinetic modeling (Kolmogorov, 1937 ▶; Johnson & Mehl, 1939 ▶; Avrami, 1939 ▶, 1940 ▶, 1941 ▶; Gualtieri, 2001 ▶), and crystallite growth has been studied, for example, by the Lifshitz–Slyzov–Wagner model describing Ostwald ripening (Lifshitz & Slyozov, 1961 ▶; Wagner, 1961 ▶) or models for oriented attachment (Xue *et al.*, 2014 ▶). These types of modeling approaches have been used extensively to obtain information on, for example, the presumed mechanisms dictating particle crystallization as well as quantitative measures, for example for rate constants and activation energies for the processes (Zhao *et al.*, 2011 ▶; Wang *et al.*, 2014 ▶; Mondloch *et al.*, 2009 ▶, 2010 ▶; Mondloch & Finke, 2011 ▶; Tyrsted *et al.*, 2010 ▶; Shields *et al.*, 2010 ▶; Jensen *et al.*, 2011 ▶; Laumann *et al.*, 2011 ▶; Eltzholtz *et al.*, 2013 ▶; Millange *et al.*, 2011 ▶; Richards *et al.*, 2011 ▶; Finney *et al.*, 2012 ▶). The present study for the first time reveals direct structural information about nanoparticle formation and growth, and the chemical processes are observed to be much more complex than assumed in the kinetics modeling. Local structural analysis in real time provides unprecedented mechanistic insight, and this may be an important step towards truly making materials by design.

## 4. Conclusion   

In summary, the solvothermal synthesis of yttria-stabilized zirconia has been revealed as consisting of three distinct structural stages. Prior to nucleation, the existence of polymeric zirconia double chains containing well defined local ordering over a length of around 10 Å have been observed. Upon nucleation, polymers fragment before clustering together into amorphous precipitates with a monoclinic-like local structure over an *r*-range of around 8 Å. The amorphous structure orders over time into the final cubic lattice structure during a bond reformation in which the structural rearrangement of the local environment for zirconium and yttrium appears equal. The study reveals the complexity of solvothermal synthesis and the need for local structural analysis for chemical understanding.

## Supplementary Material

Supporting information file. DOI: 10.1107/S2052252514006538/fc5001sup1.pdf
