# Supplementary material for: Evolution of atomic structure during nanoparticle formation
Source: IUCrJ. 2014 Apr 14;1(Pt 3):165–71. doi: 10.1107/S2052252514006538 (PMC4086431; doi:10.1107/S2052252514006538)
Supplement: Supplementary file 1 [file m-01-00165-sup1.pdf]

IUCrJ

Volume 1 (2014)

Supporting information for article:

Evolution of atomic structure during nanoparticle formation

Christoffer Tyrsted, Nina Lock, Kirsten M. Ø. Jensen, Mogens Christensen, Espen D. Bøjesen, Hermann Emerich, Gavin Vaughan, Simon J. L. Billinge and Bo B. Iversen

S1. Total X-ray Scattering

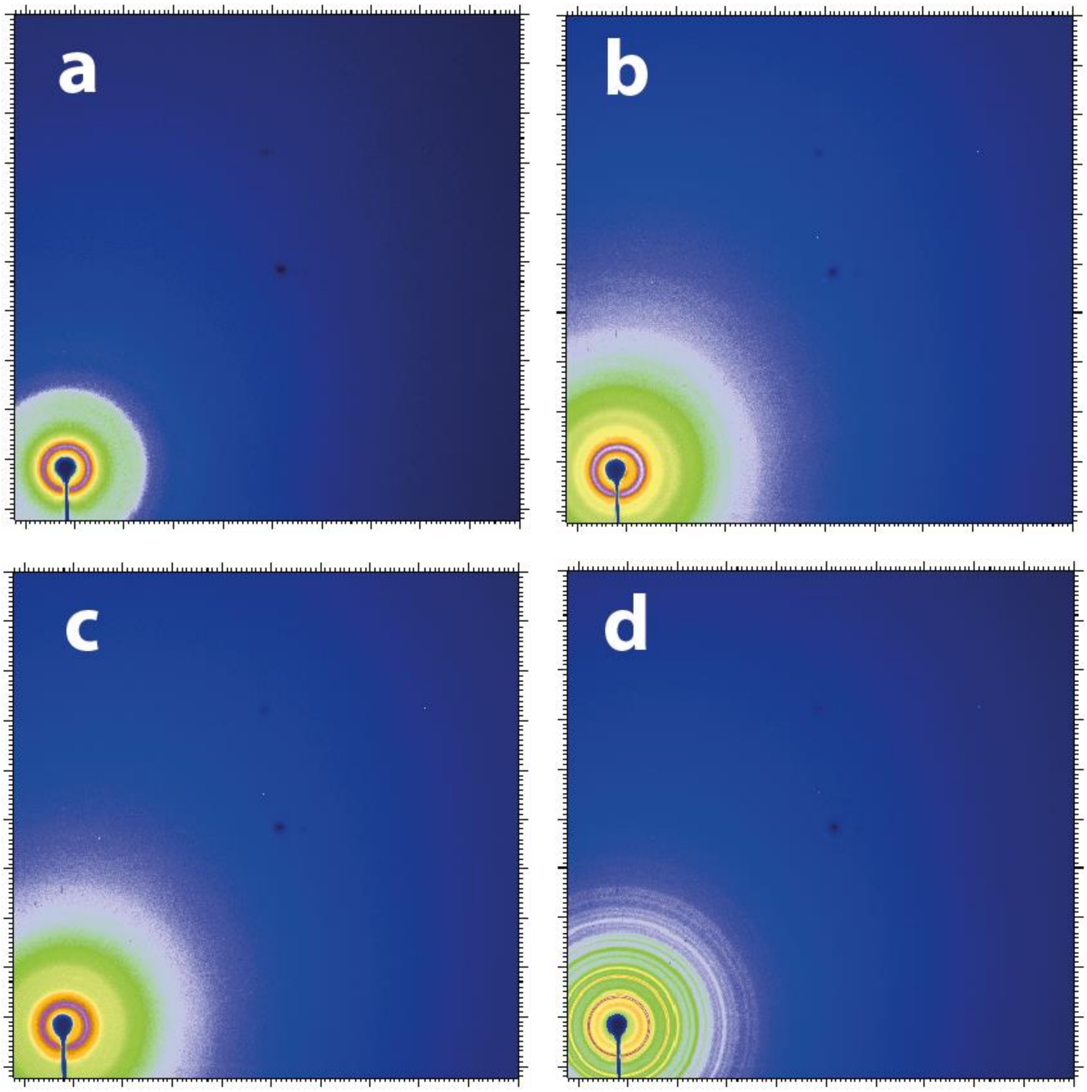

Figure S1 Unmasked detector image frames recorded for A) pure methanol, B) precursor solution, C) amorphous precipitates suspended in solution and D) nanocrystalline powder suspended in solution.

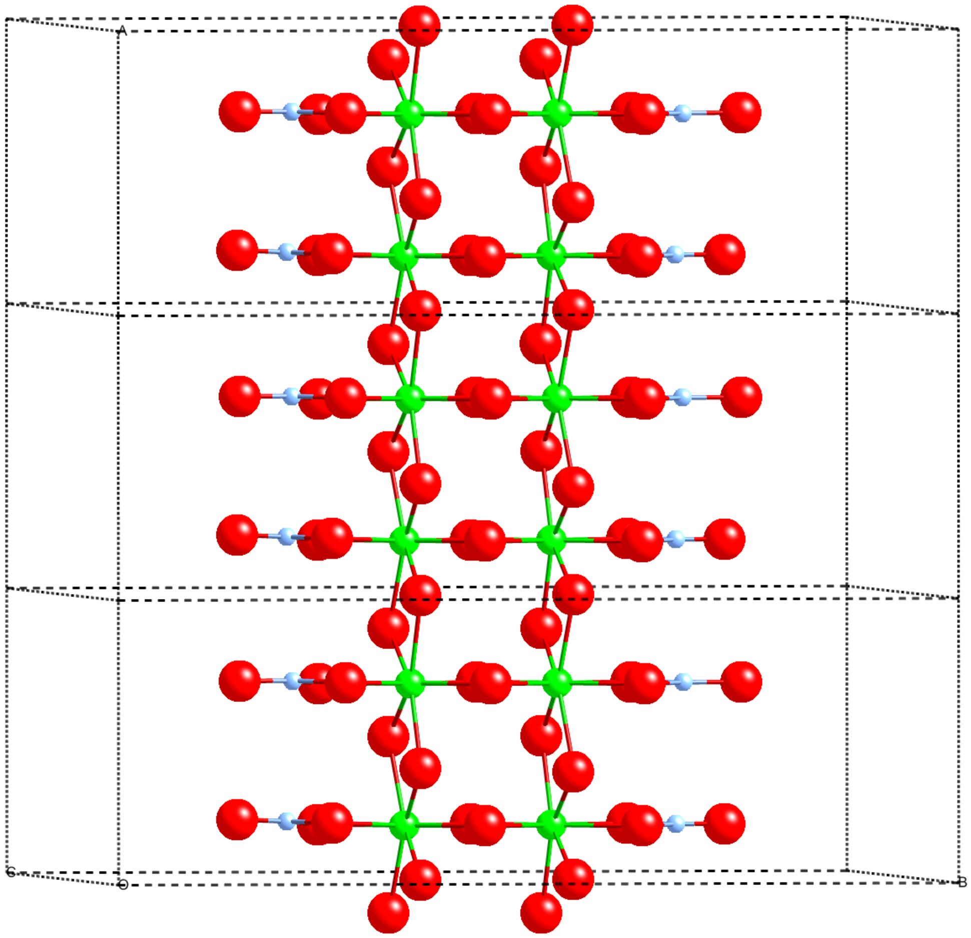

Figure S2 Structural model used for real space Rietveld refinement of precursor solution species. Zirconium (green atoms), oxygen (red atoms) and nitrogen (blue atoms).

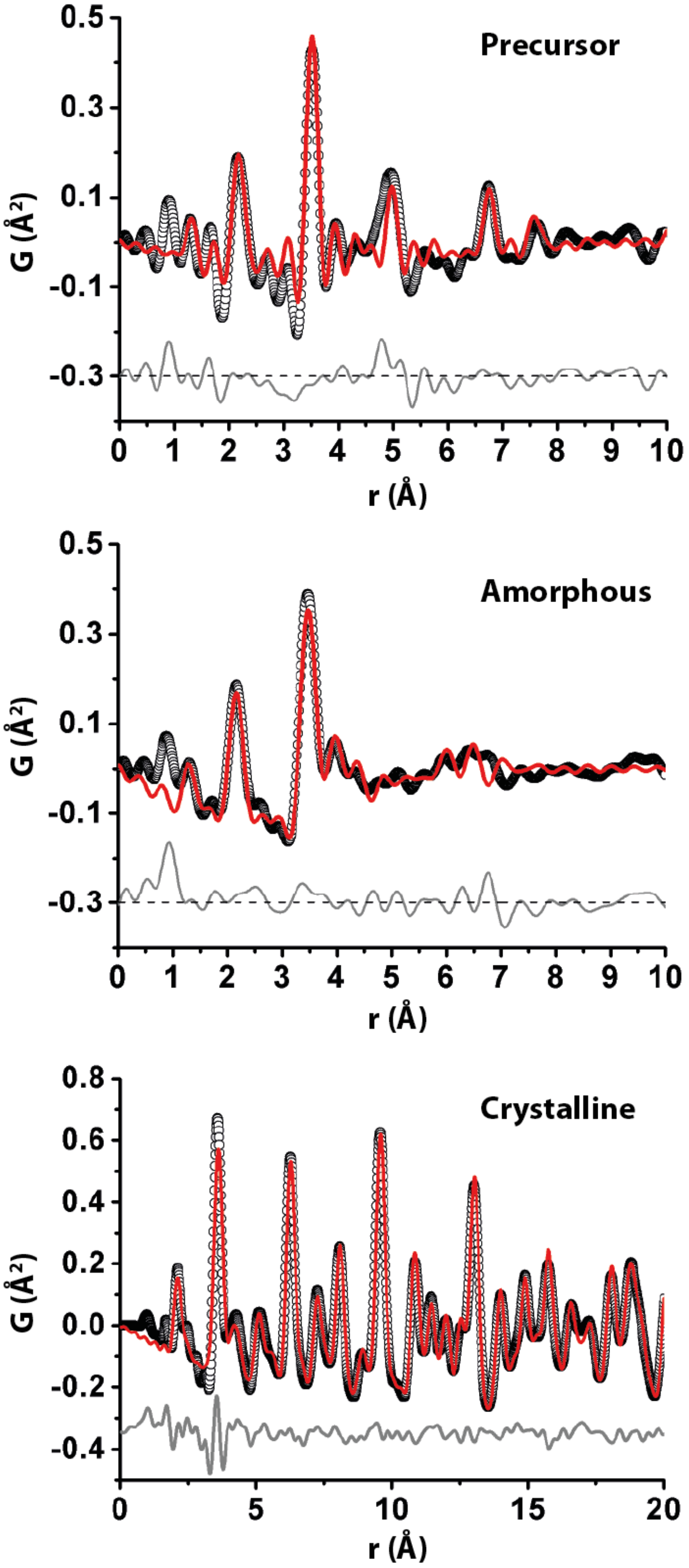

Figure S3 Real space Rietveld refinements of total scattering PDF corresponding to the three distinct structural stages; precursor species, amorphous solid and crystalline solid.

S2. X-ray Absorption Spectroscopy

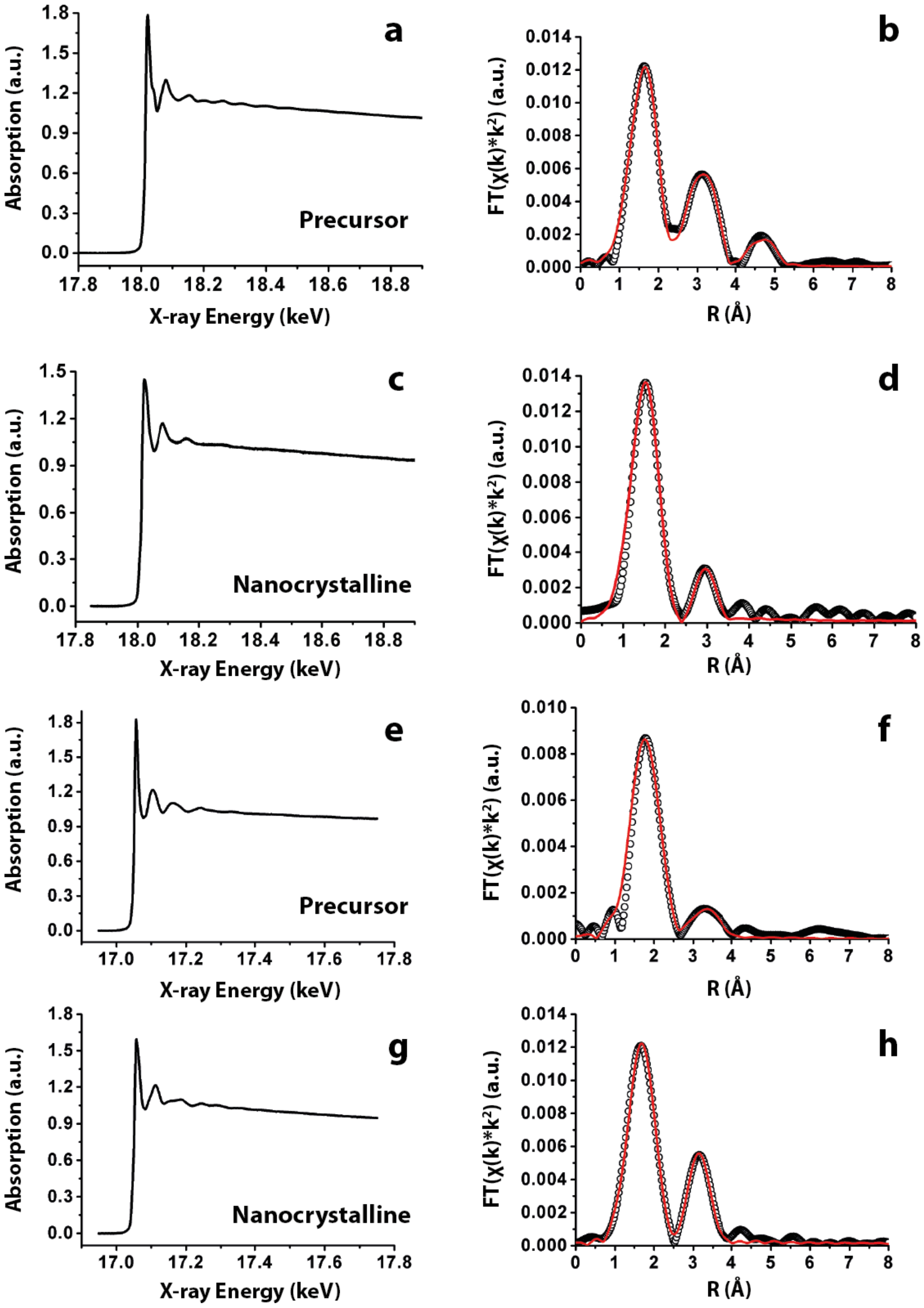

Figure S4 Absorption spectroscopy and EXAFS refinement of phase-shifted radial distribution functions for (A,B): precursor (Zr K-edge), (C,D): crystalline (Zr K-edge), (E,F): precursor (Y K-edge), (G,H): crystalline (Y K-edge)

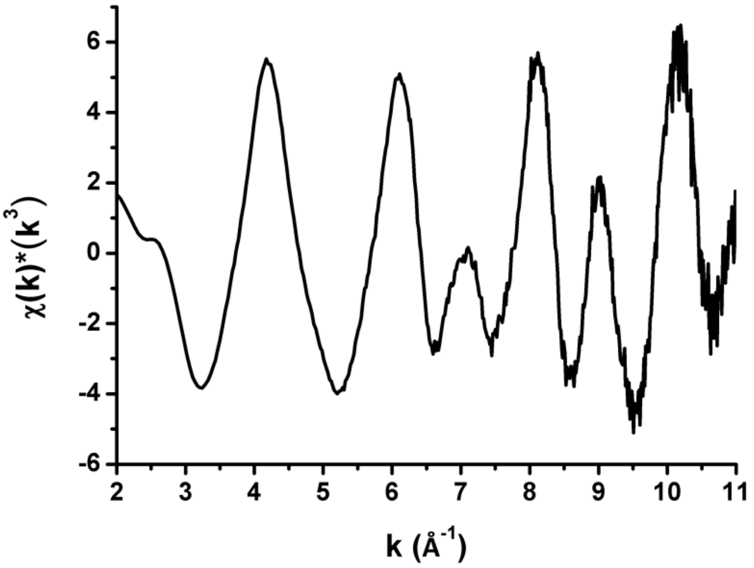

Figure S5 Isolated k^3-weighted EXAFS signal for the precursor stage, measured at the Zr K-edge.

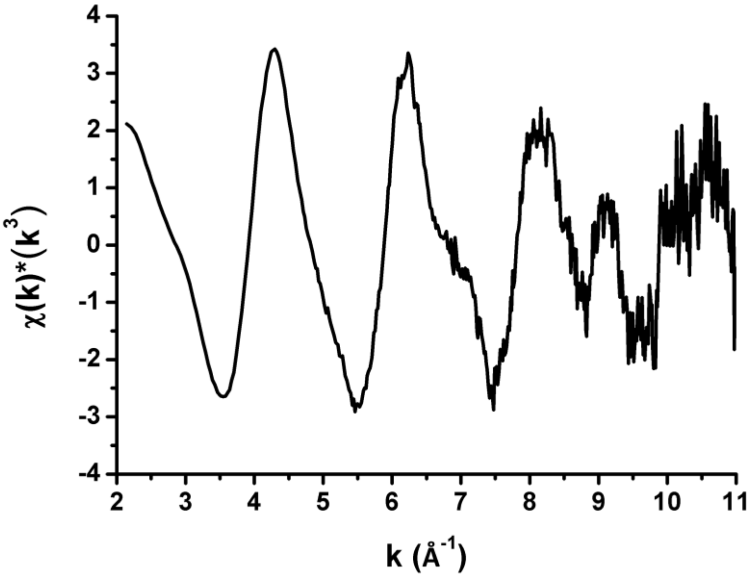

Figure S6 Isolated k^3-weighted EXAFS signal for the nanocrystalline stage, measured at the Zr K-edge.

S3. Tables

Table S1 Refinement parameters (scale factor not shown) for the crystalline stage of $Y_{0.16}Zr_{0.84}O_{1.92}$ synthesis in methanol at 275 °C for 20 minutes.

Data range	0.01 – 50 Å
Number of data points (All/Nyquist)	4999/238
Number of refined parameters	5
Spacegroup	*Fm-3m*
Qdamp	0.028945 $Å^{-1}$
R_w	0.181
a	5.16(4) Å
u_{iso}(Zr/Y)	0.011(4) $Å^2$
u_{iso}(O)	0.022(3) $Å^2$
delta2	2.1 $Å^2$
Sp-diameter	32.6(5) Å

Table S2 Refinement parameters (scale factor not shown) for the amorphous stage of $Y_{0.16}Zr_{0.84}O_{1.92}$ synthesis in methanol at 275 °C for 3 minutes.

Data range	0.01 – 10 Å
Number of data points (All/Nyquist)	999/47
Number of refined parameters	9
Spacegroup	P21/c
Qdamp	0.028945 $Å^{-1}$
R_w	0.373
a	4.97(3) Å
b	5.24(3) Å
c	5.36(3) Å
beta	97(7)°
u_{iso}(Zr)	0.002(8) $Å^2$
u_{iso}(O)	0.005(2) $Å^2$
Occ. (O)	0.86(1)
delta2	3.46 $Å^2$
Sp-diameter	8(1) Å

Atom	x-coordinate	y-coordinate	z-coordinate
Zr1	0.2823	0.0340	0.2276
Zr2	0.7344	0.9672	0.7798
Zr3	0.7344	0.5308	0.2840
Zr4	0.2823	0.4565	0.7228
O1	0.0850	0.2779	0.3919
O2	0.9108	0.6108	0.6548
O3	0.9108	0.8756	0.1177
O4	0.0850	0.1916	0.8117
O5	0.4650	0.7549	0.4789
O6	0.5577	0.2451	0.5211
O7	0.5577	0.2549	0.0211
O8	0.4650	0.7451	0.9789

Table S3 Refinement parameters (scale factor not shown) for the precursor stage of $Y_{0.16}Zr_{0.84}O_{1.92}$ synthesis in methanol at 275 ^{o}C for 0 minutes. The nitrogen atoms (N) are from the NO_3 groups (see Figure S2)

Data range	0.01 – 10 Å
Number of data points (All/Nyquist)	999/47
Number of refined parameters	5
Spacegroup	*P1*
Qdamp	0.028945 $Å^{-1}$
R_w	0.423
a	6.7(2) Å
b	20.1(3) Å
c	19.6(2) Å
alpha	90°
beta	90°
gamma	90°
u_{iso}(Zr)	0.002 $Å^2$
u_{iso}(O/N)	0.001 $Å^2$

delta2	2.1 Å^2		
Sp-diameter	10(2) Å		
Atom	x-coordinate	y-coordinate	z-coordinate
N1	0.1982	0.2714	0.5310
N2	0.6861	0.7358	0.4769
N3	0.1856	0.7341	0.5305
N4	0.6961	0.2676	0.4531
O1	0.1867	0.6829	0.5802
O2	0.6905	0.3254	0.4096
O3	0.6833	0.3072	0.5140
O4	0.9971	0.6007	0.4429
O5	0.9949	0.4183	0.4419
O6	0.3753	0.6010	0.4431
O7	0.3882	0.4182	0.4398
O8	0.4968	0.5783	0.5621
O9	0.4938	0.3961	0.5608
O10	0.8737	0.3966	0.5611
O11	0.8730	0.5783	0.5621
O12	0.1942	0.3184	0.4769
O13	0.6854	0.6811	0.5264
O14	0.6833	0.6835	0.4240
O15	0.1853	0.3156	0.5845
O16	0.6850	0.4985	0.5420
O17	0.6850	0.4985	0.4098
O18	0.1839	0.4985	0.5941
O19	0.1839	0.4985	0.4623

Table S4 EXAFS refinement results (S_0^2 = 0.83(3), E_0 = -3.1(2) eV, Residual = 10.7%) for the precursor stage (Zr K-edge) of $Y_{0.16}Zr_{0.84}O_{1.92.}$

Path	CN	*R* (Å)	σ^2 (Å^2)
Zr – O	8	2.242(8)	0.010(1)
Zr – Zr/Y	12	3.55(1)	0.012(3)

Table S5 EXAFS refinement results (S_0^2 = 0.88(2), E_0 = -2.7(3) eV, Residual = 8.5%) for the nanocrystalline stage (Zr K-edge) of $Y_{0.16}Zr_{0.84}O_{1.92}$.

Path	CN	*R* (Å)	σ^2 (Å^2)
Zr – O	8	2.19(1)	0.013(2)
Zr – Zr/Y	12	3.58(1)	0.015(2)
